# Prevalence of and Risk Factors for Nonprescription Antibiotic Use among Individuals Presenting to One Hospital in Saudi Arabia after the 2018 Executive Regulations of Health Practice Law: A Cross-Sectional Study

**DOI:** 10.3390/antibiotics10080923

**Published:** 2021-07-29

**Authors:** Ali Mohsen Al-Hazmi, Ahmed Arafa, Haytham Sheerah, Khalid Saeed Alshehri, Khalid Alwalid Alekrish, Khalid Abdullah Aleisa, Abdullah Ali Jammah, Nawaf Abdullah Alamri

**Affiliations:** 1 Health Promotion and Health Education Research, Research Chairs Program, King Saud University, Riyadh 11451, Saudi Arabia; haytham.sheerah@live.com; 2 Department of Family & Community Medicine, College of Medicine, King Saud University, Riyadh 11451, Saudi Arabia; 3 Public Health, Department of Social Medicine, Graduate School of Medicine, Osaka University, Osaka 565-0871, Japan; ahmed011172@med.bsu.edu.eg; 4 Department of Public Health, Faculty of Medicine, Beni-Suef University, Beni-Suef 62514, Egypt; 5 College of Medicine, King Saud University, Riyadh 11451, Saudi Arabia; kh0shehri@gmail.com (K.S.A.); Khalidalekrish@gmail.com (K.A.A.); Aleisa.Khaled@gmail.com (K.A.A.); Abdullahjammah@gmail.com (A.A.J.); NNAAHH18@gmail.com (N.A.A.)

**Keywords:** risk factors, nonprescription antibiotic use, Saudi Arabia

## Abstract

Antibiotic resistance is a worldwide public health emergency. Nonprescription antibiotic use is a chief cause of antibiotic resistance. The Saudi Government, as a consequence, imposed in 2018 executive regulations to prevent the distribution of antibiotics without a prescription. Herein, we aimed to investigate the prevalence of and risk factors for nonprescription antibiotic use among individuals presenting to one hospital in Saudi Arabia after enacting these regulations. This cross-sectional study was conducted on people, aged ≥18 years, who presented to the primary healthcare clinics of King Khalid University Hospital in Riyadh during the period between 1/1/2019 and 28/2/2019. Participants were asked to fill out a self-administrated questionnaire assessing their nonprescription antibiotic use during the past year in addition to sociodemographic information. Then, logistic regression analyses were performed to calculate the odds ratios (ORs) and corresponding 95% confidence intervals (CIs) for age, sex, education, and nationality of any nonprescription antibiotic use compared with no use within the past year. Out of 463 participants, 62.9% were females, 67.4% were <40 years, and 93.7% were Saudi citizens. Overall, 30.5% of participants reported nonprescription antibiotic use during the past year (19.7% one to two times and 10.8% more than two times). Male and non-Saudi participants were more likely to report any nonprescription antibiotic use, with HRs (95% CIs) of 1.99 (1.30, 3.04) and 3.81 (1.73, 8.35), respectively. The main reasons behind nonprescription antibiotic use were having previous experience with a health condition (69.2%), inaccessibility of healthcare (26.6%), and recommendation from a relative or a friend (16.1%). A major limitation of this study was that it included individuals attending one hospital. Individuals who seek medical consultation could be dissimilar to those who do not see doctors regarding nonprescription antibiotic use.

## 1. Introduction

As early as 1945, Sir Alexander Fleming, best known for discovering penicillin, warned of antibiotic abuse due to the expected increase in public demand [1]. Decades later, antibiotic abuse became a global public health crisis [2]. 

Nonprescription antibiotic use, defined as the utilization of antibiotics to treat self-diagnosed health conditions without medical consultation and prescription, is considered a major form of antibiotic abuse that can lead to antibiotic resistance [3,4,5]. Antibiotic resistance contributes to high morbidity and mortality rates and waste of public expenditure worldwide [2]. 

Previous studies showed a high prevalence of nonprescription antibiotic use in developed [6,7,8,9,10] and developing countries [11,12,13,14,15,16,17,18,19]. This high prevalence was shown to be associated with many sociodemographic characteristics including age, sex, and education level. Further, the reasons behind nonprescription antibiotic use varied from the inaccessibility of healthcare services to having a previous similar health condition [20,21]. 

Saudi Arabia was among the first countries to raise alarm against nonprescription antibiotic use. An early study, by Saeed (1988), showed that more than half of the primary healthcare patients in one clinic in Riyadh attempted the use of nonprescription medication including antibiotics [22]. Later, several studies from Saudi Arabia assessed self-medication including nonprescription antibiotic use and concluded high prevalence rates [23,24,25,26,27,28,29,30]: 86.0% among Sharjah University pharmacy students in 2012 [23], 64.8% among Taibah University students in 2015 [26], 55.2% among Buraydah Private Colleges and Qassim University students in 2015 [27], 75.2% among medical students and interns in King Abdulaziz University in 2015 [28], and 49.3% among medical students in Dammam University in 2017 [29]. However, most of these studies investigated nonprescription medication use, not specifically antibiotics; they also did not assess the risk factors for this practice and were mostly conducted on university students [23,24,25,26,27,28,29,30]. 

To curb self-medication and nonprescription antibiotic use in Saudi Arabia, the Saudi Ministry of Health imposed in 2018 provisions of the Executive Regulations of Health Practice Law, which prohibits pharmacists from dispensing drugs without a medical prescription issued by a licensed physician. According to the new regulations, no antibiotics are allowed to be sold as over-the-counter medications. The ministry warned that pharmacists who break the new provisions may face up to USD 26,600 in fines. These actions were accompanied by several activities to raise awareness among the public about the dangers of irrational use of antibiotics [31].

Given the rising consensus that nonprescription antibiotic use is a serious public health challenge that needs novel strategies for prevention, in addition to the lack of studies covering this topic in Saudi Arabia after imposing the new regulations, we, therefore, conducted this cross-sectional study to assess the prevalence of nonprescription antibiotic use among people attending the primary healthcare clinics of King Khalid University Hospital in Riyadh and investigate the risk factors for this practice and the reasons behind it.

## 2. Methods

### 2.1. Study Design, Participants, and Setting

The Saudi healthcare system has four healthcare levels. The first level is primary healthcare that provides essential curative, preventive, and promotive services. The second level consists of general and peripheral community hospitals that offer specialized diagnostic and curative services. The third level includes equipped hospitals to provide advanced diagnostic, curative, and rehabilitative services. The fourth level comprises medical sites where patients receive highly advanced health services such as stem cell transplantation and chemoimmunotherapy. Most second-, third-, and fourth-level healthcare settings provide primary healthcare services as well.

In this cross-sectional study, our eligibility criteria included: (1) people who presented to the primary healthcare clinics of King Khalid University Hospital in Riyadh during the period between 1/1/2019 and 28/2/2019 and (2) aged ≥18 years. The primary healthcare clinics of King Khalid University Hospital receive 10 to 15 patients during the working days (five days per week) between 9 a.m. and 2 p.m. Healthcare services in these clinics are provided for free to Saudi and non-Saudi people. Eligible people, regardless of their health conditions, were asked to participate in this study after seeing their physicians and receiving their medical examinations. Those who agreed to participate were asked to sign informed consent forms and complete a questionnaire. No incentives were offered for participation.

We calculated the minimum sample size using the Epi-Info version 7 Stat Calc (Center for Disease Control (CDC), World Health Organizations (WHO)) based on the following criteria: (1) population size of 999,999; (2) expected prevalence of nonprescription antibiotic use of 25%; (3) a confidence level of 95% and (4) a margin of error of 5%. However, we increased the minimum required sample size by almost 20% to enhance the statistical power of the study, avoid an unexpected low response rate, and allow us to further stratify nonprescription antibiotic users by frequency.

### 2.2. Data Collection Method

Participants were asked to fill out a paper-based self-administrated Arabic questionnaire composed of two sections. Section 1 included sociodemographic characteristics including age (18–29, 30–39, 40–49, or ≥50 years), sex (female or male), educational level (uneducated, elementary level, high school or equal, university, or postgraduate), nationality (Saudi or non-Saudi), and residence (city or village). Section 2 included a question about antibiotic use: “*during the past year, how many times have you used antibiotics without a prescription?*” The responses were “*never*”, “*one or two times*”, and “*more than two times*”. Those who reported nonprescription antibiotic use were asked “*why have you used antibiotic without prescription*?” and respondents were allowed to select one or more of the following responses: “*previous experience with the health condition*”, “*inaccessibility of healthcare*”, “*recommendation from a relative or a friend*”, and “*other unspecified reasons*”. All individuals completed the questionnaires for themselves, except for five uneducated participants, who received assistance from their companions and the data collectors. All questionnaires were finally collected by the last five authors of this study.

### 2.3. Statistical Analyses

Descriptive data in the form of frequencies and percentages were used to present the sociodemographic characteristics of the participants and the reasons behind nonprescription antibiotic use. The logistic regression analyses were performed to calculate the odds ratios (ORs) and corresponding 95% confidence intervals (CIs) for age (<40 versus ≥40 years), sex (male versus female), education (high school and lower education versus university and postgraduate), and nationality (Saudi versus non-Saudi) of (1) any nonprescription antibiotic (“*one or two times*” and “*more than two times*” compared with “*never*”) during the past year and (2) frequent nonprescription antibiotic (“*more than two times*” compared with “*never*”) during the past year. Two models of regression analyses were conducted: unadjusted and adjusted for age, sex, and education. Because of the limited frequency of village residents, the residence variable was excluded from the regression models. As a sensitivity analysis, we repeated the analysis using the same categories used in our data collection questionnaire. *p*-values < 0.05 were considered statistically significant. The Statistical Package for Social Science (SPSS) was used for analysis.

## 3. Results

Out of 512 eligible individuals, a total of 463 individuals participated in this study, with a response rate of 90.4% The main reason for nonparticipation was not having enough time. Of the 463 participants, 32.0% were aged between 18 and 29 years, 35.4% between 30 and 39 years, 17.9% between 40 and 49 years, and 14.7% ≥50 years. Almost two-thirds of participants were females and most of them were Saudi citizens. Overall, 30.5% of participants reported nonprescription antibiotic use in the past year (19.7% one to two times and 10.8% more than two times) (Table 1).

In the unadjusted model, male and non-Saudi participants were more likely to report any nonprescription antibiotic use, with HRs (95% CIs) of 2.04 (1.36, 3.05) and 3.54 (1.64, 7.63), respectively. Adjusting for other factors did not change the results, with HRs (95% CIs) of 1.99 (1.30, 3.04) and 3.81 (1.73, 8.35), respectively (Table 2). Similarly, male and non-Saudi participants were more likely to report frequent nonprescription antibiotic use, with HRs (95% CIs) of 2.13 (1.17, 3.88) and 3.52 (1.26, 9.86), and these findings did not change in the adjusted model, with values of 2.27 (1.21, 4.27) and 3.86 (1.35, 11.05), respectively (Table 3).

When we repeated the analysis, whether for any versus never or frequent versus never nonprescription antibiotic use, using the same categories as in (Table 1), the results did not significantly change (Supplementary Tables S1 and S2).

**Figure 1:**
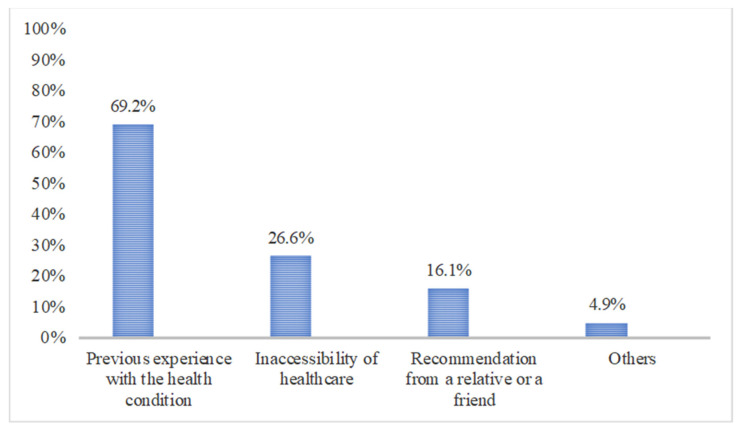
Reasons behind nonprescription antibiotic use.

The reasons behind nonprescription antibiotic use were having previous experience with the health condition (69.2%), inaccessibility of healthcare (26.6%), a recommendation from a relative or a friend (16.1%), and other unspecified reasons (4.9%) (Figure 1).

## 4. Discussion

This study indicated that the prevalence of nonprescription antibiotic use during the past year among people who presented to the primary healthcare clinics of King Khalid University Hospital in Riyadh between 1/1/2019 and 28/2/2019 was 30.5%. Male and non-Saudi participants reported more nonprescription antibiotic use than female and Saudi participants. Having previous experience with the health condition and inaccessibility of healthcare were the major reasons behind nonprescription antibiotic use.

Our results differed from a previous study conducted on 519 people who presented to the same primary healthcare clinics in Riyadh in 2015 [30]. Although the sociodemographic characteristics of participants of the previous study were almost the same as ours, their study showed a significantly higher prevalence of nonprescription antibiotic use during the year before data collection (40.8%). The 10.3% difference in the results between the two studies could be partly attributed to the Executive Regulations of Health Practice Law that was issued in 2018 to regulate the dispensing of antibiotics. The law stressed that pharmacists must require a medical prescription; otherwise, they may have to pay hefty fines [31]. Moreover, the Saudi Ministry of Health has launched several awareness campaigns to warn the public against the adverse outcomes of nonprescription antibiotic use [31]. These campaigns might have also contributed to reducing the prevalence of nonprescription antibiotic use.

It is quite difficult to compare our results with those emerging from other international studies because of the wide variations in the sociodemographic characteristics across the studies, which may affect the prevalence, differences in the study designs, inconsistencies in defining nonprescription antibiotic use, and differences in the periods during which the prevalence of nonprescription antibiotic use was assessed [6,7,8,9,10,11,12,13,14,15,16,17,18,19]. However, a systematic review of 117 relevant articles representing 35 communities from five continents showed that the prevalence of nonprescription antibiotic use ranged between 19% and 100%, with lower rates in Northern Europe and North America, where antibiotics are largely restricted to prescription-only use [3].

Of note, our results indicated that male participants tended to practice nonprescription antibiotic use more than female ones. This finding agreed with the previous study from Riyadh [30]. Compared to males, females consult their doctors, on average, more frequently and are more likely to follow health recommendations and regulations [32,33]; therefore, it should not be unexpected that they are less likely to use medications without prescription. Moreover, non-Saudi participants reported significantly more nonprescription antibiotic use than Saudi participants, which agreed with the previous study [30]. This finding can be explained by the inaccessibility of low-priced healthcare services to non-Saudi people in Saudi Arabia or it could reflect the practices in the countries they came from. Although the participants of our study had already received healthcare at primary healthcare clinics, most of the non-Saudi people who needed further investigations or expensive medications including antibiotics had to pay most of the expenses. Unfortunately, we have no data on the exact nationalities of participants, which could have helped in explaining this association. We, therefore, believe that more research should be done to understand the variations in nonprescription antibiotic use across different nationalities residing in Saudi Arabia.

In agreement with the previous Riyadh study and other studies conducted on Arab populations [12,13,14,15,30], the main reasons behind nonprescription antibiotic use were having previous experience with the health condition, inaccessibility of healthcare, and recommendation from a relative or a friend. As long as these reasons have not changed since the previous Riyadh study [30], national programs should focus on eliminating the reasons behind nonprescription antibiotic use. Awareness programs should focus on the dangers of using leftover drugs from a previous disease. More effort should be made to make healthcare accessible, especially for non-Saudi residents. 

It should be noted that this study posed several limitations that should be addressed. First, this is a unicentric study that was conducted on people presenting to the primary healthcare clinics of one university hospital. Perhaps individuals who do not go to healthcare centers are more likely to self-medicate than those who visit the doctor at some point. Second, although we investigated the primary healthcare clinics during the past year of data collection only, the possibility of recall bias cannot be entirely excluded. It could be speculated that patients with severe infections probably remember better that they had an infectious condition and that an antibiotic was prescribed, while those with milder conditions were probably more likely to forget that they had taken an antibiotic. Third, the cross-sectional design of this study did not allow us to assess the trends of nonprescription antibiotic use throughout the past years. Fourth, some sociodemographic characteristics that could be associated with nonprescription antibiotic use, such as annual income, occupation, medical condition, and the exact nationalities of non-Saudi participants, were not studied. Fifth, many sociodemographic questions and questions about the reasons behind nonprescription antibiotic use were somewhat restrictive. We believe that a qualitative study might have provided more comprehensive information. Sixth, it is not clear from the study whether participants used newly purchased or leftover antibiotics. However, since this study was conducted one year after implementing the law, it is highly probable that nonprescription antibiotic use in our study included mostly newly purchased antibiotics. Seventh, we had no data about the types of antibiotics that were used without prescription and for which conditions. 

In conclusion, this study showed that nonprescription antibiotic use was not uncommon among people in Saudi Arabia. Being male or non-Saudi was a predictor of nonprescription antibiotic use. People in Saudi Arabia resorted to nonprescription antibiotic use if they had previous experience with the health condition or in the case of the inaccessibility of healthcare. Compared to a previous similar study in Riyadh [30], a 10% decrease in nonprescription antibiotic use could be noticed, which reflects the possible impacts of the laws set and awareness campaigns provided by the Saudi Ministry of Health to regulate the dispensing of antibiotics [31]. 

Future research should correlate antibiotic resistance and risk factors for nonprescription antibiotic use in Saudi Arabia. Qualitative research to figure out the general population’s, doctors’, and pharmacists’ perceptions about nonprescription antibiotic use in Saudi Arabia and the newly imposed law should be considered. Moreover, studying the prevalence of and risk factors for nonprescription antibiotic use among non-Saudi residents should be considered. More awareness programs should be initiated to educate people about the importance of avoiding antibiotic abuse, especially nonprescription antibiotic use. These programs should be tailored to be suitable for the sociocultural characteristics of people living in Saudi Arabia.

## Figures and Tables

**Table 1:** Sociodemographic characteristics of participants and their nonprescription antibiotic use.

Characteristics		*N* = 463 (%)
Age	18–29 years	148 (32.0)
	30–39 years	164 (35.4)
	40–49 years	83 (17.9)
	≥50 years	68 (14.7)
Sex	Female	291 (62.9)
	Male	172 (37.1)
Education	Uneducated	5 (1.1)
	Elementary	43 (9.3)
	High or equal	124 (26.8)
	University	235 (50.8)
	Postgraduate	56 (12.0)
Nationality	Saudi	434 (93.7)
	Non-Saudi	29 (6.3)
Residence	City	451 (97.4)
	Village	12 (2.6)
Nonprescription antibiotic use in the past year	Never	322 (69.5)
	1–2 times	91 (19.7)
	>2 times	50 (10.8)

**Table 2:** Association between sociodemographic characteristics of participants and any nonprescription antibiotic use.

Characteristics		Never *n* (%)	Any *n* (%)	Unadjusted OR (95% CI)	*p*-Value	Adjusted OR (95% CI)	*p*-Value
Age	<40 years	221 (70.8)	91 (29.2)	1	0.387	1	0.657
	≥40 years	101 (66.9)	50 (33.1)	1.20 (0.79, 1.83)		1.11 (0.71, 1.74)	
Sex	Female	219 (75.3)	72 (24.7)	1	0.001	1	0.002
	Male	103 (59.9)	69 (40.1)	2.04 (1.36, 3.05)		1.99 (1.30, 3.04)	
Education	High school or lower	129 (75.0)	43 (25.0)	1	0.051	1	0.084
	University or higher	193 (66.3)	98 (33.7)	1.52 (1.00, 2.32)		1.48 (0.95, 2.31)	
Nationality	Saudi	310 (71.4)	124 (28.6)	1	0.001	1	0.001
	Non-Saudi	12 (41.4)	17 (58.6)	3.54 (1.64, 7.63)		3.81 (1.73, 8.35)	

Regression models were adjusted for age, sex, education, and nationality.

**Table 3:** Association between sociodemographic characteristics of participants and frequent nonprescription antibiotic use (>2 times).

Characteristics		Never *n* (%)	>2 Times *n* (%)	Unadjusted OR (95% CI)	*p*-Value	Adjusted OR (95% CI)	*p*-Value
Age	<40 years	221 (86.7)	34 (13.3)	1	0.928	1	0.739
	≥40 years	101 (86.3)	16 (13.7)	1.03 (0.54, 1.95)		0.89 (0.45, 1.76)	
Sex	Female	219 (89.8)	25 (10.2)	1	0.014	1	0.011
	Male	103 (80.5)	25 (19.5)	2.13 (1.17, 3.88)		2.27 (1.21, 4.27)	
Education	High school or lower	129 (87.2)	19 (12.8)	1	0.841	1	0.900
	University or higher	193 (86.2)	31 (13.8)	1.09 (0.59, 2.01)		1.04 (0.55, 1.98)	
Nationality	Saudi	310 (87.6)	44 (12.4)	1	0.017	1	0.012
	Non-Saudi	12 (66.7)	6 (33.3)	3.52 (1.26, 9.86)		3.86 (1.35, 11.05)	

Regression models were adjusted for age, sex, education, and nationality.

## Data Availability

Available upon request.

## Supplementary Materials

The following are available online at https://www.mdpi.com/article/10.3390/antibiotics10080923/s1, Table S1. Association between sociodemographic characteristics of participants and any nonprescription antibiotic use, Table S2. Association between sociodemographic characteristics of participants and frequent nonprescription antibiotic use (>2 times).

## References

[1] Spellberg B., Gilbert D.N. (2014). The Future of Antibiotics and Resistance: A Tribute to a Career of Leadership by John Bartlett. Clin. Infect. Dis..

[2] Aslam B., Wang W., Arshad M.I., Khurshid M., Muzammil S., Rasool M.H., Nisar M.A., Alvi R.F., Aslam M.A., Qamar M.U. (2018). Antibiotic resistance: A rundown of a global crisis. Infect. Drug Resist..

[3] Morgan D.J., Okeke I., Laxminarayan R., Perencevich E., Weisenberg S. (2011). Non-prescription antimicrobial use worldwide: A systematic review. Lancet Infect. Dis..

[4] Contopoulos-Ioannidis D.G., Koliofoti I.D., Koutroumpa I.C., Giannakakis I.A., Ioannidis J.P.A. (2001). Pathways for Inappropriate Dispensing of Antibiotics for Rhinosinusitis: A Randomized Trial. Clin. Infect. Dis..

[5] Llor C., Bjerrum L. (2014). Antimicrobial resistance: Risk associated with antibiotic overuse and initiatives to reduce the problem. Ther. Adv. Drug Saf..

[6] Zoorob R., Grigoryan L., Nash S., Trautner B.W. (2016). Nonprescription Antimicrobial Use in a Primary Care Population in the United States. Antimicrob. Agents Chemother..

[7] Berzanskyte A., Valinteliene R., Haaijer-Ruskamp F.M., Gurevicius R., Grigoryan L. (2006). SELF-MEDICATION WITH ANTIBIOTICS IN LITHUANIA. Int. J. Occup. Med. Environ. Health.

[8] Skliros E., Merkouris P., Papazafiropoulou A., Gikas A., Matzouranis G., Papafragos C., Tsakanikas I., Zarbala I., Vasibosis A., Stamataki P. (2010). Self-medication with antibiotics in rural population in Greece: A cross-sectional multicenter study. BMC Fam. Pr..

[9] Ramalhinho I., Cordeiro C., Cavaco A., Cabrita J. (2014). Assessing determinants of self-medication with antibiotics among Portuguese people in the Algarve Region. Int. J. Clin. Pharm..

[10] Grigoryan L., Haaijer-Ruskamp F.M., Burgerhof J., Mechtler R., Deschepper R., Tambic-Andrasevic A., Andrajati R., Monnet D.L., Cunney R., Di Matteo A. (2006). Self-medication with Antimicrobial Drugs in Europe. Emerg. Infect. Dis..

[11] Widayati A., Suryawati S., De Crespigny C., E Hiller J. (2011). Self medication with antibiotics in Yogyakarta City Indonesia: A cross sectional population-based survey. BMC Res. Notes.

[12] Al-Azzam S.I., Al-Husein B.A., Alzoubi F., Masadeh M.M., Al-Horani M.A. (2007). Self-Medication with Antibiotics in Jordanian Population. Int. J. Occup. Med. Environ. Health.

[13] Shehadeh M., Suaifan G., Darwish R., Wazaify M., Zaru L., Alja’Fari S. (2012). Knowledge, attitudes and behavior regarding antibiotics use and misuse among adults in the community of Jordan. A pilot study. Saudi Pharm. J..

[14] Awad A., Eltayeb I., Matowe L., Thalib L. (2005). Self-medication with antibiotics and antimalarials in the community of Khartoum State, Sudan. J. Pharm. Pharm. Sci..

[15] Barah F., Goncalves V., Barah F., Goncalves V. (2010). Antibiotic use and knowledge in the community in Kalamoon, Syrian Arab Republic: A cross-sectional study. East. Mediterr. Health J..

[16] Shankar P.R., Partha P., Shenoy N. (2002). Self-medication and non-doctor prescription practices in Pokhara valley, Western Nepal: A questionnaire-based study. BMC Fam. Pr..

[17] Biswas M., Roy M.N., Manik I.N., Hossain S., Alam Tapu S.M.T., Moniruzzaman M., Sultana S. (2014). Self medicated antibiotics in Bangladesh: A cross-sectional health survey conducted in the Rajshahi City. BMC Public Health.

[18] Ocan M., Obuku E.A., Bwanga F., Akena D., Richard S., Ogwal-Okeng J., Obua C. (2015). Household antimicrobial self-medication: A systematic review and meta-analysis of the burden, risk factors and outcomes in developing countries. BMC Public Health.

[19] Aslam A., Gajdács M., Zin C.S., Ab Rahman N.S., Ahmed S.I., Zafar M.Z., Jamshed S. (2020). Evidence of the Practice of Self-Medication with Antibiotics among the Lay Public in Low- and Middle-Income Countries: A Scoping Review. Antibiotics.

[20] Lescure D., Paget J., Schellevis F., Van Dijk L. (2018). Determinants of Self-Medication With Antibiotics in European and Anglo-Saxon Countries: A Systematic Review of the Literature. Front. Public Health.

[21] Schmiege D., Evers M., Kistemann T., Falkenberg T. (2020). What drives antibiotic use in the community? A systematic review of determinants in the human outpatient sector. Int. J. Hyg. Environ. Health.

[22] Saeed A.A. (1988). Self-medication among primary care patients in Farazdak clinic in Riyadh. Soc. Sci. Med..

[23] Sharif S.I., Ibrahim O.M., Mouslli L., Waisi R. (2012). Evaluation of self-medication among pharmacy students. Am. J. Pharmacol. Toxicol..

[24] Eissa A.T. (2013). Knowledge, Attitudes and Practices towards Medication Use among Health Care Students in King Saud University. Int. J. Med Stud..

[25] Al-Worafi Y.A., Long C., Saeed M., Alkhoshaiban A. (2014). Perception of self-medication among university students in Saudi Arabia. Arch. Pharm. Pr..

[26] Aljaouni M.E., Hafiz A.A., Alalawi H.H., Alahmadi G.A., AlKhawaja I. (2015). Self-medication practice among medical and non-medical students at Taibah University, Madinah, Saudi Arabia. Int. J. Acad. Sci. Res..

[27] Karim M.A.S., Adnan S.K.M. (2015). Evaluation of Self-Medication Practices and Awareness among Students in Al Qassim Region of Saudi Arabia. Clin. Pharmacol. Biopharm..

[28] Ibrahim N.K., AlAmoudi B.M., Baamer W.O., Al-Raddadi R.M. (2014). Self-medication with analgesics among medical students and interns in King Abdulaziz University, Jeddah, Saudi Arabia. Pak. J. Med. Sci..

[29] Albusalih F.A., Naqvi A.A., Ahmad R., Ahmad N. (2017). Prevalence of Self-Medication among Students of Pharmacy and Medicine Colleges of a Public Sector University in Dammam City, Saudi Arabia. Pharmacy.

[30] Al-Qahtani A.A., Al-Qahtani M.A., Amin H.S., Alshahrani A.M., Alghamdi H.A., Althwayee M.S., Alzahrani A.A. (2018). Self-medication with antibiotics in a primary care setting in King Khalid University Hospital, Riyadh, Saudi Arabia. J. Fam. Community Med..

[31] MOH News MOH Warns against Selling Antibiotics without Prescription. https://www.moh.gov.sa/en/Ministry/MediaCenter/News/Pages/news-2018-04-17-004.aspx.

[32] Hunt K., Adamson J., Hewitt C., Nazareth I. (2011). Do women consult more than men? A review of gender and consultation for back pain and headache. J. Heal. Serv. Res. Policy.

[33] Vlassoff C. (2007). Gender Differences in Determinants and Consequences of Health and Illness. J. Health Popul. Nutr..

