# Supplementary material for: Prevalence of and Risk Factors for Nonprescription Antibiotic Use among Individuals Presenting to One Hospital in Saudi Arabia after the 2018 Executive Regulations of Health Practice Law: A Cross-Sectional Study
Source: Antibiotics (Basel). 2021 Jul 29;10(8):923. doi: 10.3390/antibiotics10080923 (PMC8388660; doi:10.3390/antibiotics10080923)
Supplement: Supplementary file 1 [file antibiotics-10-00923-s001.zip › antibiotics-1257839-supplementary.pdf]

Supplementary Table S1. Association between sociodemographic characteristics of participants and any nonprescription antibiotic use.

	B	S.E.	Wald	df	Sig.	Exp(B)	95% CI for EXP(B)	
							Lower	Upper
Age			2.298	3	.513			
Age(1)	.281	.263	1.140	1	.286	1.325	.791	2.219
Age(2)	.317	.317	1.001	1	.317	1.374	.738	2.558
Age(3)	-.083	.362	.052	1	.819	.921	.453	1.870
Gender(1)	.732	.223	10.793	1	.001	2.079	1.343	3.218
Education			8.171	4	.086			
Education(1)	-.199	.981	.041	1	.839	.819	.120	5.608
Education(2)	-1.160	.959	1.462	1	.227	.314	.048	2.055
Education(3)	-.510	.944	.292	1	.589	.601	.094	3.823
Education(4)	-.588	.979	.361	1	.548	.555	.082	3.784
Nationality(1)	1.391	.412	11.402	1	.001	4.018	1.792	9.006
Constant	-.732	.946	.599	1	.439	.481		

a. Variable(s) entered: Age, Gender, Education, Nationality.

Supplementary Table S2. Association between sociodemographic characteristics of participants and frequent nonprescription antibiotic use (>2 times).

	B	S.E.	Wald	df	Sig.	Exp(B)	95% CI for EXP(B)	
							Lower	Upper
Age			4.432	3	.218			
Age(1)	.718	.408	3.099	1	.078	2.050	.922	4.556
Age(2)	.477	.493	.936	1	.333	1.611	.613	4.229
Age(3)	-.107	.581	.034	1	.854	.899	.288	2.807
Gender(1)	.817	.330	6.118	1	.013	2.264	1.185	4.325
Education			2.647	4	.619			
Education(1)	-.430	1.268	.115	1	.735	.651	.054	7.816
Education(2)	-1.206	1.226	.967	1	.325	.299	.027	3.312
Education(3)	-.996	1.205	.684	1	.408	.369	.035	3.916
Education(4)	-1.173	1.272	.850	1	.356	.309	.026	3.745
Nationality(1)	1.482	.548	7.300	1	.007	4.401	1.502	12.896
Constant	-1.666	1.210	1.896	1	.168	.189		

a. Variable(s) entered: Age, Gender, Education, Nationality.
